# Association between left ventricular ejection fraction, mortality and use of mechanical circulatory support in patients with non-ischaemic cardiogenic shock

**DOI:** 10.1007/s00392-023-02332-y

**Published:** 2023-11-20

**Authors:** Jonas Sundermeyer, Caroline Kellner, Benedikt N. Beer, Lisa Besch, Angela Dettling, Letizia Fausta Bertoldi, Stefan Blankenberg, Jeroen Dauw, Zouhir Dindane, Dennis Eckner, Ingo Eitel, Tobias Graf, Patrick Horn, Joanna Jozwiak-Nozdrzykowska, Paulus Kirchhof, Stefan Kluge, Axel Linke, Ulf Landmesser, Peter Luedike, Enzo Lüsebrink, Nicolas Majunke, Norman Mangner, Octavian Maniuc, Sven Möbius Winkler, Peter Nordbeck, Martin Orban, Federico Pappalardo, Matthias Pauschinger, Michal Pazdernik, Alastair Proudfoot, Matthew Kelham, Tienush Rassaf, Clemens Scherer, Paul Christian Schulze, Robert H. G. Schwinger, Carsten Skurk, Marek Sramko, Guido Tavazzi, Holger Thiele, Luca Villanova, Nuccia Morici, Ralf Westenfeld, Ephraim B. Winzer, Dirk Westermann, Benedikt Schrage

**Affiliations:** 1 Department of Cardiology, University Heart and Vascular Center Hamburg, Martinistr. 52, 20251 Hamburg, Germany; 2 German Center for Cardiovascular Research (DZHK), Partner Site Hamburg/Kiel/Lübeck, Hamburg, Germany; 3 Cardio Center, Humanitas Clinical and Research Center—IRCCS, Rozzano, Milan Italy; 4 Department of Cardiology, Paracelsus Medical University Nürnberg, Nuremberg, Germany; 5 University Heart Center Lübeck, University Hospital Schleswig-Holstein, Lübeck, Germany; 6 Division of Cardiology, Pulmonology and Vascular Medicine, Medical Faculty, University Duesseldorf, Duesseldorf, Germany; 7 Department of Intensive Care Medicine, University Medical Center Hamburg-Eppendorf, Hamburg, Germany; 8 Department of Cardiology, Charité Universitätsmedizin Berlin, Campus Benjamin Franklin, Berlin, Germany; 9 Department of Cardiology and Vascular Medicine, West German Heart and Vascular Center, University Hospital Essen, Essen, Germany; 10 Department of Medicine I, University Hospital, LMU Munich, Munich, Germany; 11 Department of Internal Medicine I, University Hospital Würzburg, Würzburg, Germany; 12 Department of Internal Medicine I, University Hospital Jena, Jena, Germany; 13 Dept Cardiothoracic and Vascular Anesthesia and Intensive Care, AO SS Antonio E Biagio E Cesare Arrigo, Alessandria, Italy; 14 Department of Cardiology, IKEM, Prague, Czech Republic; 15 Department of Perioperative Medicine, St. Bartholomew’s Hospital, London, UK; 16 Medizinische Klinik II, Kliniken Nordoberpfalz AG, Weiden, Germany; 17 Department of Internal Medicine and Cardiology, Heart Center Leipzig at University of Leipzig and Leipzig Heart Institute, Leipzig, Germany; 18 Unità Di Cure Intensive Cardiologiche and De Gasperis Cardio-Center, ASST Grande Ospedale Metropolitano Niguarda, Milan, Italy; 19 IRCCS S. Maria Nascente-Fondazione Don Carlo Gnocchi ONLUS, Milan, Italy; 20 Herzzentrum Dresden, Technische Universität Dresden, Dresden, Germany; 21 Department of Cardiology and Angiology, University Heart Center Freiburg-Bad Krozingen, Freiburg, Germany; 22 Department of Cardiology, AZ Sint-Lucas, Ghent, Belgium

**Keywords:** Cardiogenic shock, Non-ischaemic, Left ventricular ejection fraction, Mechanical circulatory support

## Abstract

**Background:**

Currently, use of mechanical circulatory support (MCS) in non-ischaemic cardiogenic shock (CS) is predominantly guided by shock-specific markers, and not by markers of cardiac function. We hypothesise that left ventricular ejection fraction (LVEF) can identify patients with a higher likelihood to benefit from MCS and thus help to optimise their expected benefit.

**Methods:**

Patients with non-ischaemic CS and available data on LVEF from 16 tertiary-care centres in five countries were analysed. Cox regression models were fitted to evaluate the association between LVEF and mortality, as well as the interaction between LVEF, MCS use and mortality.

**Results:**

*N* = 807 patients were analysed: mean age 63 [interquartile range (IQR) 51.5–72.0] years, 601 (74.5%) male, lactate 4.9 (IQR 2.6–8.5) mmol/l, LVEF 20 (IQR 15–30) %. Lower LVEF was more frequent amongst patients with more severe CS, and MCS was more likely used in patients with lower LVEF. There was no association between LVEF and 30-day mortality risk in the overall study cohort. However, there was a significant interaction between MCS use and LVEF, indicating a lower 30-day mortality risk with MCS use in patients with LVEF ≤ 20% (hazard ratio 0.72, 95% confidence interval 0.51–1.02 for LVEF ≤ 20% vs. hazard ratio 1.31, 95% confidence interval 0.85–2.01 for LVEF > 20%, interaction-*p* = 0.017).

**Conclusion:**

This retrospective study may indicate a lower mortality risk with MCS use only in patients with severely reduced LVEF. This may propose the inclusion of LVEF as an adjunctive parameter for MCS decision-making in non-ischaemic CS, aiming to optimise the benefit–risk ratio.

**Graphical abstract:**

Impact of left ventricular ejection fraction on mortality and use of mechanical circulatory support in non-ischaemic cardiogenic shock. Hazard ratio for 30-day mortality across the LVEF continuum, adjusted for age, sex, SCAI shock stage, worst value of lactate and pH within 6 h, prior resuscitation and mechanical ventilation during the index shock event. LVEF: Left ventricular ejection fraction; MCS: Mechanical circulatory support; HR: Hazard ratio; CI: Confidence interval.

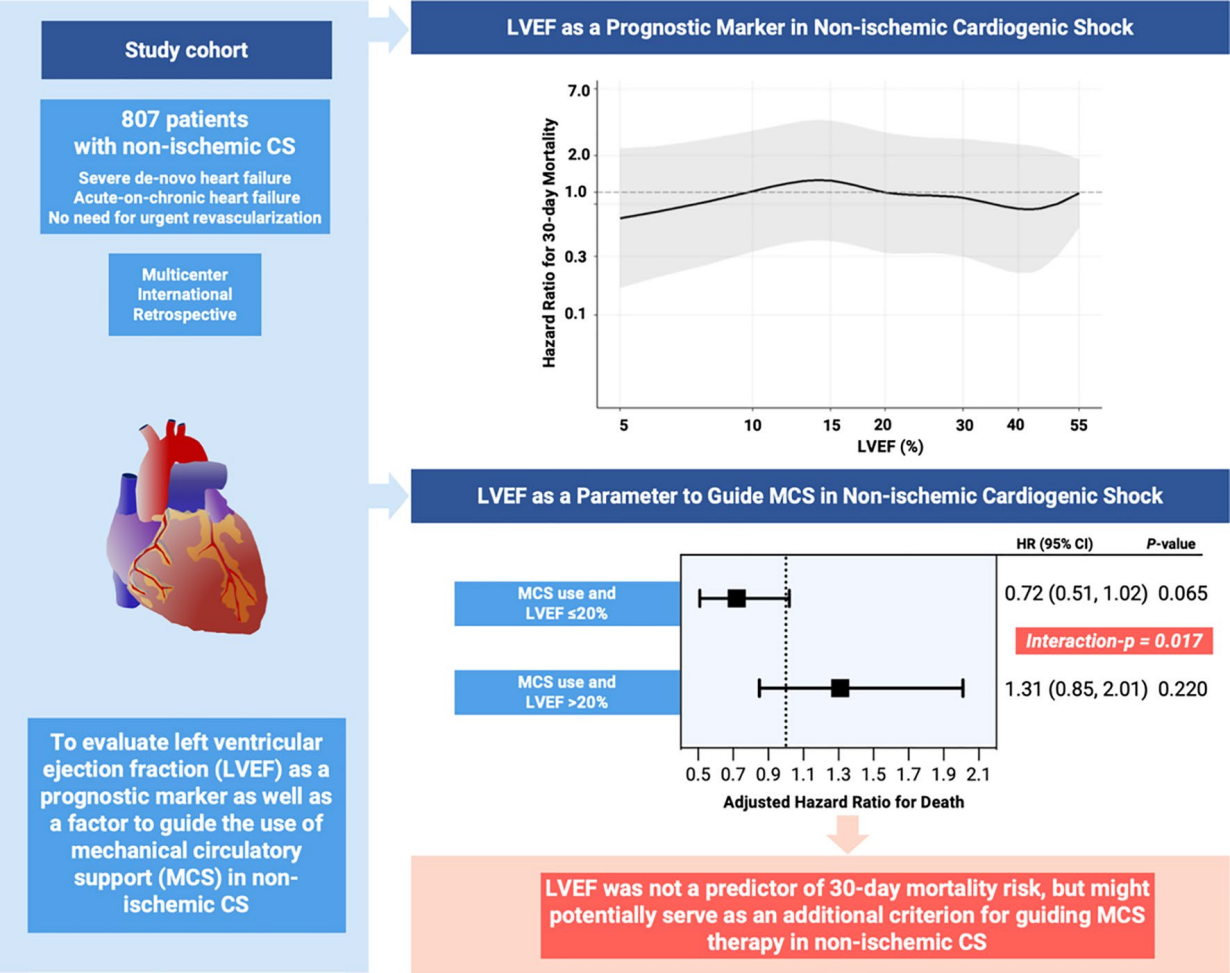

**Supplementary Information:**

The online version contains supplementary material available at 10.1007/s00392-023-02332-y.

## Introduction

Cardiogenic shock (CS) is a serious pathological condition with acute deterioration of cardiac output, leading to life-threatening hypo-perfusion of end-organs, and can be caused by a wide range of cardiovascular diseases [1–3]. In the last decade, a convincing reduction in mortality has only been achieved in CS caused by an acute myocardial infarction (AMI) through early revascularization of the culprit lesion [4–6]. However, despite intensive research efforts in the past decades, no additional treatment strategies have demonstrated mortality reduction, specifically in patients with non-ischaemic CS, such that short-term mortality is still over 50% [7, 8].

Approximately half of all patients with CS have a non-ischaemic cause, with mortality rates at least as equivalent to AMI-CS [7, 9, 10]. The heterogeneity of the underlying pathology makes this group a clinical challenge, particularly in terms of clinical assessment and targeted CS treatments. Mechanical circulatory support (MCS) could improve outcome in CS regardless of its underlying cause by providing cardiac output support until native heart recovery or as a bridge to more definitive strategies or decision [15]. However, aside from the recently initiated UNLOAD-ECMO trial [11], current randomised controlled trials (RCT) for MCS continue to focus exclusively on the AMI-CS patient cohort (DanGer-SHOCK for pLVADs, ECLS-SHOCK and ANCHOR for VA-ECMO) [12–14]. There is no randomised evidence for the targeted use of inotropes, vasopressors or MCS in the large group of patients with non-ischaemic CS. In addition, the use of MCS devices is also associated with notable complications which may impact outcomes [7, 15–19]. Hence, further research on the use of MCS devices in patients with non-ischaemic CS is an unmet need.

Using quantitative measures of cardiac function seems to be reasonable both for facilitating early assessment of CS and guiding the appropriate use of MCS devices. Left ventricular ejection fraction (LVEF) can be obtained quickly and non-invasively from transthoracic echocardiography (TTE) and conceptually may be a useful quantitative measure of severity or prognosis, given its association with shock severity [20]. As extension of this, it is plausible that LVEF may identify a cohort of patients most likely to benefit from MCS in patients with non-ischaemic CS. [2, 3, 14]

Hence, the aim of this study was to evaluate whether LVEF can be used as a prognostic marker for early assessment as well as a factor to guide the use of MCS devices in non-ischaemic CS.

## Methods

This study was carried out in accordance with the Declaration of Helsinki and accredited by local ethics committees. The main ethics committee renounced the need for an informed consent as this was a retrospective analysis and only entirely anonymized data was analysed. The data supporting the results of this study are available from the corresponding author upon reasonable request.

### Study design

In this study, patients with non-ischaemic CS treated with or without MCS between 01 January 2010 and 31 December 2020 from 16 tertiary care centres in five countries were collected retrospectively (NCT03313687). Patients were eligible for this study if they presented with CS according to the Society for Cardiovascular Angiography & Interventions (SCAI) CS definition, as retrospectively determined by the local investigators upon reviewing the available case data. Non-ischaemic shock caused by severe decompensation in patients with known heart failure (severe acute-on-chronic heart failure) or unknown heart failure (severe de novo heart failure) was used as the main inclusion criterion.

Patients were not eligible for the study if they presented with acute myocardial infarction or had need for urgent coronary revascularization (irrespective of feasibility); had CS primarily caused by right heart failure (e.g. acute pulmonary embolism); had ECMO-assisted resuscitation; had a post-cardiotomy CS or had other disease which limits life expectancy to below 6 months.

If patients were treated with MCS, the index event was defined as the time of implantation of the first device. If patients were not treated with MCS, baseline was defined as admission to the hospital for out-patients or admission to the intensive care unit for in-patients. For variables determining shock severity, e.g. lactate and pH, the worst value within 6 h before until 6 h after this index event (e.g. a 12-h window) was recorded.

From this registry, only patients with available LVEF measurements obtained via TTE at the time of the index event according to international guidelines were included in this analysis. [21–24]

Statistical analyses

Continuous variables are shown as median (25th percentile, 75th percentile) and analysed using Mann–Whitney test. For binary variables, absolute and relative frequencies are given and comparisons were made using the Fisher`s exact test. Two-level Joint Modelling Multiple Imputation was used to handle missing data. The used clusters were known/unknown history of heart failure. Parameters used for imputation were 20 imputed data sets, 5000 iterations between two successive imputations and 5000 burns in iterations (R package jomo [25]). Table 1 indicates the variables used for the imputation. The following analyses were calculated in imputed data sets.

**Table 1:** Characteristics for the overall cohort and divided by LVEF > 20% vs. ≤ 20%

	All (*N* = 807)	Missing data (%)	LVEF > 20% (*N* = 351)	LVEF ≤ 20% (*N* = 456)	*p* value
*Demographics*					
Age, years	63.0 (51.5, 72.0)	0	66.0 (55.0, 76.0)	60.0 (49.0, 70.0)	< 0.0001
Male sex	601 (74.5)	0	238 (67.8)	363 (79.6)	0.00017
*Previous heart failure status*					
Ischaemic cardiomyopathy*	221 (33.3)	17.7	96 (35.7)	125 (31.6	0.31
Previous heart failure hospitalizations, n	2.0 (1.0, 3.0)	59.1	1.0 (1.0, 3.0)	2.0 (1.0, 3.0)	0.041
*Previous heart failure treatment*					
Beta-blocker (No)	329 (42.1)	3.1	137 (40.3)	192 (43.4)	0.38
Renin–angiotensin system inhibitors (No)	380 (48.5)	3	162 (47.5)	218 (49.3)	0.67
Mineralocorticoid receptor antagonists (No)	492 (62.8)	2.9	229 (67.2)	263 (59.4)	0.026
Implantable cardioverter defibrillator	269 (33.4)	< 1	89 (25.4)	180 (39.6)	< 0.0001
Cardiac resynchronization therapy	110 (13.7)	1.8	33 (9.4)	77 (16.9)	0.0026
*Comorbidities*					
Atrial fibrillation	353 (44.3)	1.2	148 (42.4)	205 (45.8)	0.35
Diabetes mellitus	213 (26.7)	1	107 (30.5)	106 (23.7)	0.036
Arterial hypertension	432 (54.2)	1.2	220 (63.0)	212 (47.3)	< 0.0001
Body mass index, kg/m^2^	26.4 (23.3, 30.5)	3.7	27.0 (23.7, 31.2)	26.1 (23.1, 29.5)	0.051
Prior revascularization	187 (24.5)	< 1	85 (26.0)	102 (23.4)	0.44
Any intervention for peripheral artery disease	48 (6.0)	25	22 (6.4)	26 (5.8)	0.77
*Clinical presentation*					
Systolic blood pressure, mmHg (worst value within 6 h)*	82.0 (71.0, 91.0)	1.5	85.0 (72.0, 95.0)	80.0 (70, 90)	0.0093
Diastolic blood pressure, mmHg (worst value within 6 h)*	50.0 (40.0, 59.8)	2.1	50.0 (40.0, 59.5)	50.0 (42.0, 59.5)	0.12
Vasopressor use	697 (86.5)	< 1	301 (86.0)	396 (86.8)	0.76
Heart rate, bpm (worst value within 6 h)	100.0 (78.0, 120.0)	1.5	90.0 (72.0, 114.5)	102 (80, 128)	< 0.0001
Lactate, mmol/l (worst value within 6 h)*	5.1 (2.7, 8.6)	6.8	4.7 (2.7, 8.4)	5.3 (2.8, 8.9)	0.18
pH (worst value within 6 h)*	7.3 (7.2, 7.4)	3.6	7.3 (7.2, 7.4)	7.3 (7.2, 7.4)	0.13
Prior cardiac arrest*	277 (34.5)	< 1	137 (39.3)	140 (30.9)	0.016
Duration of cardiac arrest, min	10.0 (0, 25.0)	56.3	10.0 (1.5, 30.0)	7.0 (0, 20.0)	0.0081
Mechanical ventilation*	507(64.4)	2.5	233 (67.9)	274 (61.7)	0.072
Horowitz index (worst value within 6 h)	201.5 (109.5, 297.0)	27.5	180.0 (95.0, 293.0)	219.8 (121.3, 300.0)	0.0088
Creatinine, mg/dl (worst value within 6 h)	1.7 (1.3, 2.6)	1.5	1.7 (1.2, 2.6)	1.8 (1.3, 2.7)	0.16
eGFR (ml/min)	37.5 (22.6, 60.1)	1.5	37.5 (22.6, 60.1)	37.6 (23.4, 57.4	0.76
SCAI cardiogenic shock class*					
B	114 (14.7)		62 (18.4)	52 (11.8)	0.011
C	294 (37.8)		134 (39.8)	160 (36.4)	0.37
D	204 (26.3)		85 (25.2)	119 (27.0)	0.62
E	165 (21.2)		56 (16.6)	109 (24.8)	0.0061
*Use of mechanical circulatory support*					
VA-ECMO	144 (17.8)	0	41 (11.7)	103 (22.6)	< 0.0001
Impella	133 (16.5)	0	67 (19.1)	66 (14.5)	0.085
Impella + VA-ECMO	83 (10.3)	0	26 (7.4)	57 (12.5)	0.019

Continuous variables are shown as a median (25th, 75th percentile), binary variables as absolute and relative frequencies, the *p* value given is calculated for continuous variables by Mann–Whitney test or binary variables by Fisher`s exact test. Variables marked with * were included in the multiple imputation model. LVEF, left ventricular ejection fraction; SCAI: Society for Cardiovascular Angiography & Intervention; VA-ECMO, veno-arterial extracorporeal membrane oxygenation

Multivariable mixed effects logistic regression models with centre as a random intercept were fitted in order to investigate patient characteristics (demographics, clinical characteristics, comorbidities, heart failure treatments, index event parameter) independently associated with LVEF dichotomized by median (20), adjusted for age, sex, SCAI class, lactate, prior cardiopulmonary resuscitation, mechanical ventilation and pH.

Survival curves were produced using the Kaplan–Meier method. The number of individuals at risk was given and groups were compared using log-rank test. None of the relevant variables for estimation of mortality rate were imputed, so crude mortality rates for 30-day mortality were estimated by the reverse Kaplan–Meier estimator in original data.

To assess the association between LVEF (as a continuous logarithmic variable and as a binary variable dichotomized by LVEF ≤ 20% vs. > 20%) and 30-day mortality, cohort stratified Cox proportional hazard regression models were fitted, adjusted for age, sex, SCAI class, lactate, prior cardiopulmonary resuscitation, mechanical ventilation and pH. To allow possible non-linearities in the association of LEVF with time-to-event, the previous models were modified modelling LVEF using natural cubic splines. Plots were produced to examine the shape of the association. As a sensitivity analysis, given the heterogeneity of the LVEF measurements between cohorts, the respective LVEF median value per centre was calculated, and a Cox regression model for 30-day mortality was fitted using LVEF dichotomized by this centre-specific median, also adjusted for the above described variables.

Linear mixed models with centre as a random intercept were used to identify the most important predictors for continuous LVEF, adjusted for the above described variables.

To evaluate the impact of LVEF on the association between MCS use and all-cause mortality (e.g. to assess whether MCS use would be associated with mortality only in patients with or without lower LVEF), Cox regression models with an interaction term for MCS use and LVEF were used, adjusted for the same variables described above.

All analyses were performed with R statistical software version 4.1.2. A *p* value below 0.05 was considered as statistically significant.

## Results

### Study cohort

Of 1030 patients with non-ischaemic CS in the overall study cohort, 223 patients were excluded due to missing data on LVEF at the index event, leaving a total of 807 patients in this study. Baseline characteristics for the overall cohort dichotomized by the median LVEF > 20% vs. ≤ 20% are shown in Table 1.

The median age of all patients was 63 [interquartile range (IQR) 51.5–72.0] years and 601 (74.5%) were male. Overall, 432 (54.2%) patients had arterial hypertension, 213 (26.7%) diabetes mellitus and 353 (44.3%) patients had a history of atrial fibrillation. Of 807 patients, 486 (60.2%) had an acute-on-chronic heart failure as the underlying cause of CS and 221 (32.7%) patients had a prior history of ischaemic cardiomyopathy (but no need for urgent coronary revascularization or no acute myocardial infarction during the shock index event).

At the index event, 507 (64.4%) patients were on mechanical ventilation, with a Horowitz index (PaO2/FiO2) of 200.5 (IQR 108.5–296), and 277 (34.5%) patients had a prior cardiac arrest. The baseline pH value was 7.31 (IQR 7.20–7.40) and the baseline lactate was 4.9 (IQR 2.6–8.5) mmol/l, systolic blood pressure 81.0 (IQR 70.0–90.0) mmHg and diastolic blood pressure 49.0 (39–58.8) mmHg.

### Left ventricular ejection fraction and cardiogenic shock severity

The median baseline LVEF of the study cohort was 20 (IQR 15–30) %. Patients with a severely reduced LVEF (≤ 20%) more frequently had a lower systolic blood pressure [80 (IQR 70–90) vs. 85 (IQR 72–95) mmHg, *p* < 0.01], higher heart rate [102 (IQR 80–128) vs. 90 (IQR 72–114.5) bpm, *p* < 0.01], numerically higher baseline lactate level [5.3 (IQR 2.8–8.9) vs. 4.7 (IQR 2.7–8.4) mmol/l, *p* = 0.18], less frequently had a history of ischaemic cardiomyopathy (31.6% vs. 35.1%, *p* = 0.31), but more frequently had a history of heart failure (63.6% vs. 55.8%, *p* < 0.04).

**Fig. 1:**
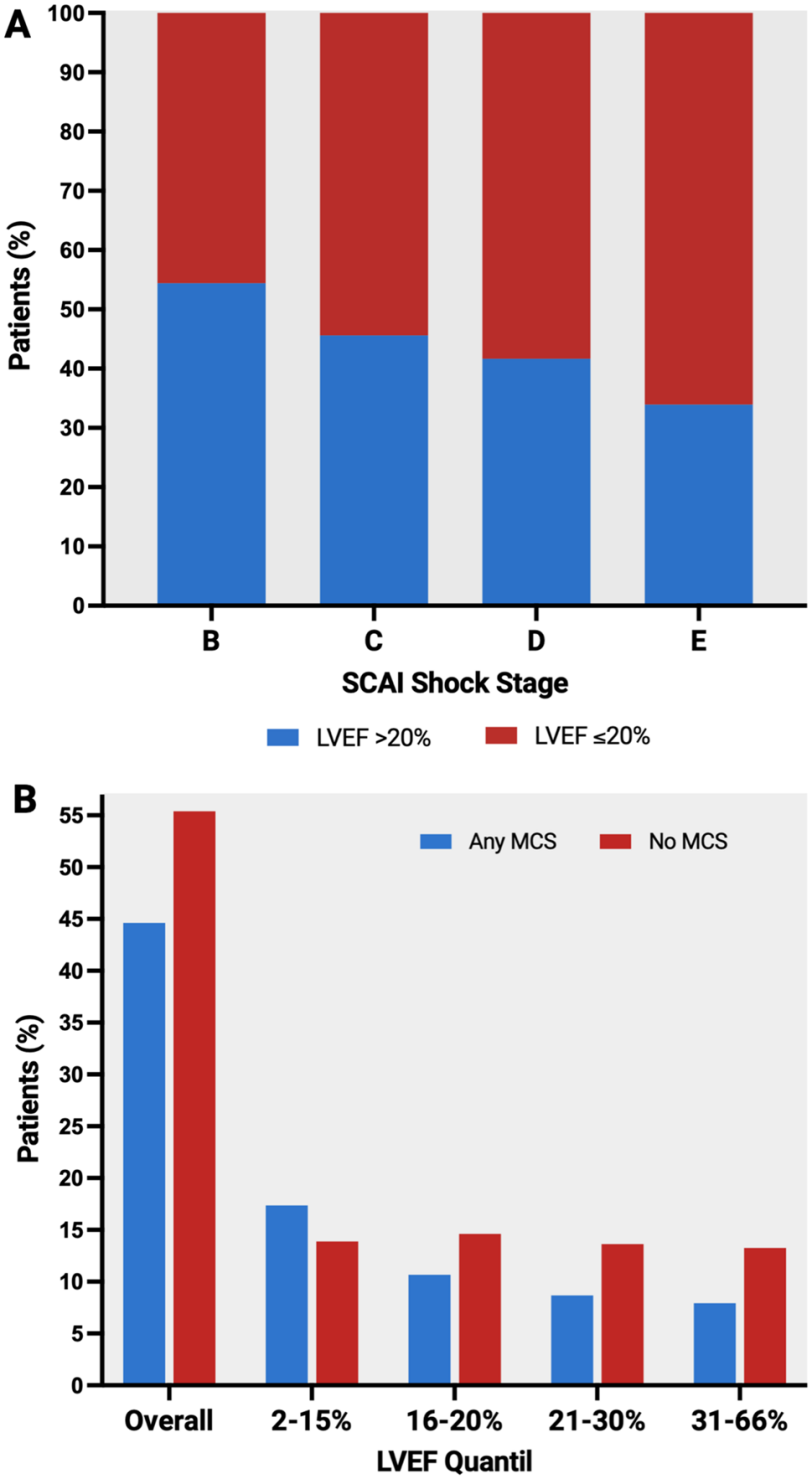
Distribution of LVEF across SCAI Shock Stages and use of mechanical circulatory support across different LVEF levels. (**A**) Distribution of LVEF > 20% versus ≤ 20% across SCAI Shock Stages. (**B**) Mechanical circulatory support use across LVEF Quantiles. LVEF, Left ventricular ejection fraction; SCAI, Society for Cardiovascular Angiography & Intervention

Overall, a trend towards lower LVEF with increasing CS severity, as indicated by SCAI CS stage, was observed (Fig. 1a). Patients presenting with CS in SCAI class C had 54.4% severely reduced LVEF (≤ 20%), whilst as many as 66.1% of patients with SCAI class E had a severely reduced LVEF. Even after adjustment for relevant confounders, this trend persisted, with severely reduced LVEF being significantly associated with a higher likelihood of worse SCAI CS stage [SCAI CS class B vs. C, odds ratio (OR) 1.63, 95% confidence interval (CI) 0.99–2.68; SCAI CS class B vs. D, OR 2.67, 95% CI 1.5–4.77; SCAI CS class B vs. E, OR 3.88, 95% CI 2.02–7.47].

#### Left ventricular ejection fraction and mortality

**Fig. 2:**
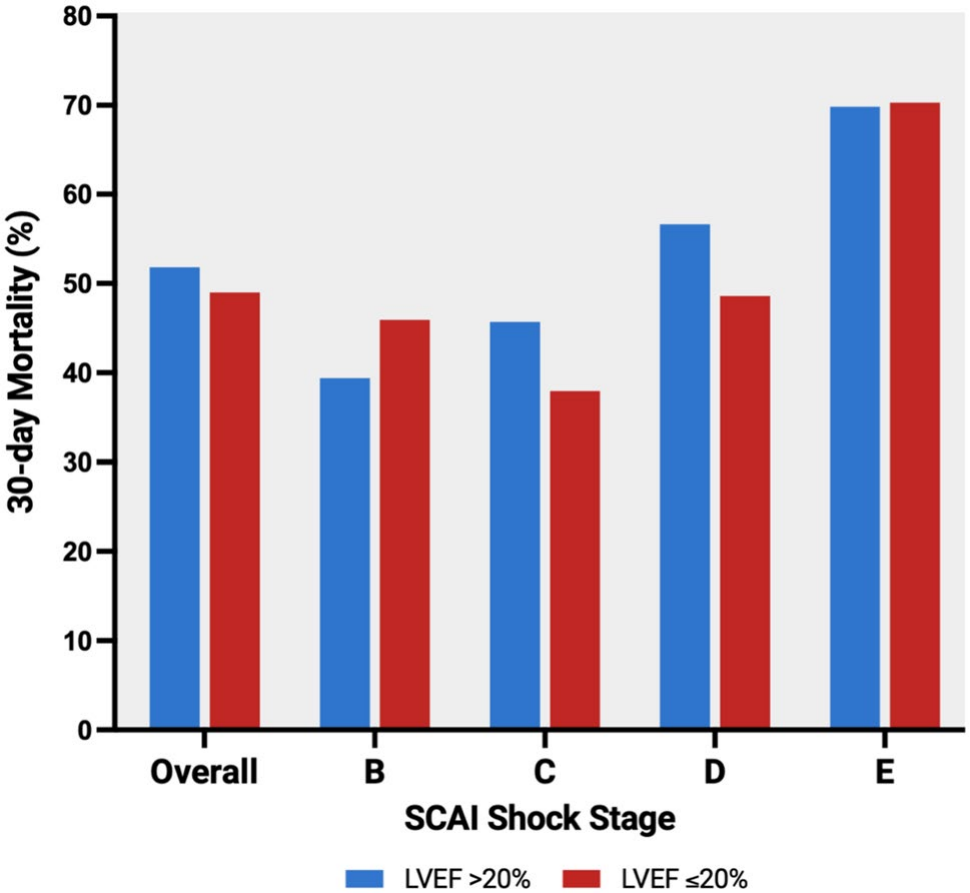
LVEF and 30-day mortality in patients with non-ischaemic cardiogenic shock. 30-day Mortality in SCAI shock stages in patients with non-ischaemic shock and LVEF > 20% versus ≤ 20%. LVEF, Left ventricular ejection fraction; SCAI, Society for Cardiovascular Angiography & Intervention

In the study cohort, 346 patients died during a follow-up period of 30 days, resulting in a crude overall mortality rate of 50.04%. 149 (42.4%) of 351 patients with LVEF > 20% died within 30 days, resulting in a mortality rate of 51.82%. In patients with a severely reduced LVEF ≤ 20%, 197 (43.2%) of 456 died within 30 days, resulting in a mortality rate of 48.99% (mortality rate for 30-day mortality in different SCAI shock stages is shown in Fig. 2***,*** Kaplan–Meier curves of the study cohort comparing LVEF > 20% versus ≤ 20% is shown in Supplementary Fig. 1).

In patients with non-ischaemic CS, there was no significant association between LVEF and 30-day mortality risk [hazard ratio (HR) 1.04, 95% CI 0.82–1.31 if LVEF was considered as a logarithmized continuous variable; HR 1.20, 95% CI 0.93–1.53 if LVEF was considered as a categorical variable with ≤ 20% vs. > 20%], even after adjustment for relevant confounders, such as age, sex, SCAI class, lactate, pH, prior resuscitation and mechanical ventilation (Graphical abstract).

In the sensitivity analysis, using a centre-specific median value for LVEF to determine higher vs. lower LVEF instead of the cohort-specific LVEF median of 20%, there was also no significant association between LVEF and 30-day mortality (LVEF > vs. ≤ centre-specific median, HR 1.21, 95% CI 0.95–1.53, **Supplementary Fig. 2**).

#### Left ventricular ejection fraction and use of mechanical circulatory support

Amongst patients with non-ischaemic CS included in this analysis, 697 (86%) patients were treated with inotropes/vasopressors; and 360 (44.6%) patients were treated with and 447 (55.4%) without MCS: 133 (16.5%) patients were treated with pLVAD (Impella®), 144 (17.8%) patients were treated with VA-ECMO, and 83 (10.3%) with both devices (no treatment with intra-aortic balloon pumps).

Baseline characteristics indicated that MCS was more frequently used in patients with severely reduced LVEF ≤ 20% (Table 1; Fig. 1b). When assessing the likelihood of MCS use in patients with higher vs. lower LVEF, and even after adjustment for age, sex, SCAI class, lactate and pH, prior resuscitation and mechanical ventilation, MCS use was more likely in patients with severely reduced LVEF (OR 1.75, 95% CI 1.14–2.68, *p*-value = 0.009, if LVEF was dichotomized by the median LVEF > 20% vs. ≤ 20%; beta 3.72, 95% CI 1.79–5.66, *p*-value < 0.001, if LVEF was considered as a continuous variable).

#### Left ventricular ejection fraction, mechanical circulatory support and mortality

In this study of patients with non-ischaemic CS, a significant interaction was observed between MCS use and LVEF, indicating a lower 30-day mortality risk for patients with MCS and LVEF ≤ 20% vs. MCS use and LVEF > 20% (HR 0.72, 95% CI 0.51–1.02 for LVEF ≤ 20% and MCS use vs. HR 1.31, 95% CI 0.85–2.01 for LVEF > 20% and MCS use, interaction-*p* = 0.017; Graphical abstract).

These results were consistent in the sensitivity analysis, using a centre-specific instead of a cohort-specific median value for LVEF, even after adjustment for age, sex, SCAI class, lactate and pH, prior resuscitation and mechanical ventilation.

## Discussion

In this retrospective, multicenter, international registry of patients with non-ischaemic CS, lower LVEF was associated with higher CS severity, but was not associated with an increased risk of 30-day mortality. However, there was a significant interaction between MCS use and severely reduced LVEF, indicating a lower mortality risk in patients with a severely reduced LVEF and treated with MCS vs. those not treated with MCS. These results suggest that severely reduced LVEF, such as LVEF ≤ 20%, could potentially serve as an additional parameter to consider when guiding the use of MCS devices in non-ischaemic CS.

### LVEF, shock severity and mortality in patients with non-ischaemic cardiogenic shock

Recent studies have indicated that nearly half of all CS cases have a non-ischaemic aetiology [9, 26]. Whilst in CS caused by acute myocardial infarction, a relevant reduction in mortality risk can be achieved by early revascularization of the culprit artery, no such risk-reducing intervention exists for non-ischaemic CS [4–6, 27]. MCS devices target the haemodynamic culprit of non-ischaemic CS and could be beneficial treatments, but are also associated with a high risk of complications. Better identification of patients with non-ischaemic CS who might benefit from MCS devices is desirable to optimise any benefit–risk ratio. We sought to evaluate if LVEF, which can be easily and rapidly measured by TTE even in the acute setting of CS, could help to guide the use of MCS in non-ischaemic CS.

Interestingly, in this large retrospective, multicenter, international study cohort, lower LVEF was associated with higher CS severity, but, after adjustment for factors reflecting CS severity, not with 30-day mortality risk. This is in contrast to studies on CS caused by acute myocardial infarction. In a sub-study of the SHould we emergently revascularize Occluded Coronaries for cardiogenic shocK (SHOCK) trial, LVEF < 28% was an independent predictor of 30-day and 1-year mortality [28]. In the CardShock study, LVEF < 40% was independently associated with increased short-term mortality in patients irrespective of CS aetiology; however, the proportion of patients with non-ischaemic CS in this study was low, introducing a high risk of bias [29]. Several observations might explain this discrepancy. The difference between the results of the present study and the various AMI-CS studies could be due to less rigorous adjustment for shock severity in previous studies as compared to this study [28, 29]. In addition, the different CS subtypes (AMI-CS vs. non-ischaemic CS) differ in their pathogenesis, comorbidity burden and most importantly treatment. Revascularization of the culprit artery in patients with AMI-CS can improve not only the survival but also LV function as a surrogate for therapeutic success. Persistently severely reduced LVEF in AMI-CS patients may therefore indicate treatment failure or suboptimal revascularization outcome. On the other hand, non-ischaemic CS is a heterogeneous condition with various subtypes and underlying pathophysiologies (e.g. acute-on-chronic HF-related CS vs. de novo HF-related CS). In some patients, preexisting LVEF depression is common, and recovery may be limited to the pre-existing LVEF after treatment. Therefore, changes in LVEF may not necessarily correlate with treatment success and improved clinical outcomes in patients with non-ischaemic CS. It is also noticeable that previous AMI-CS studies frequently examined patients with an LVEF > 30%. In contrast, the median LVEF amongst patients with non-ischaemic CS included for this analysis was lower (20%). Lastly, our findings might extend those of a previous study assessing TTE for risk prediction in the cardiac intensive care unit. In this study, measures of LV function were more useful for mortality risk stratification in patients with lower SCAI shock stages (A to C), as compared to patients with higher SCAI shock stages as observed on our cohort [20].

### Impact of LVEF on use of mechanical circulatory support in non-ischaemic cardiogenic shock

Non-ischaemic CS can be caused by a variety of triggers which either cause or aggravate a pre-existing ventricular dysfunction [9]. Most prior RCTs have excluded patients with non-ischaemic CS. Therefore there is currently no evidence-based therapy for non-ischaemic CS [12, 13, 30]. Catecholamines are frequently used to support cardiac function, but their effects are limited, and they may be associated with worse outcome. A recent study reported that increasing vasopressor requirements in patients with CS was independently associated with mortality risk [31]. Furthermore, a RCT comparing dopamine and norepinephrine in the management of CS showed no significant differences in mortality risk between the two study arms [32]. This study also demonstrated an increased risk of arrhythmias with dopamine use [32]. Even when comparing milrinone and dobutamine in treatment of CS, no significant advantage of milrinone over dobutamine in terms of efficacy and safety was found [41]. Despite an absence of compelling evidence for their use, and some association with harm, the use of inotropes including short-acting catecholamines continues to form part of international guidelines as a bridge to MCS in unstable or deteriorating patients [1, 33, 34]. Aside from catecholamines, MCS may be used for the treatment of non-ischaemic CS, but the evidence supporting this is yet scarce [1]. Also, MCS use is associated with a high risk of complications, so that selecting the right patients for this approach is crucial to optimise the benefit-risk-ratio. [3, 7, 15–19, 35]

In this study, LVEF was not associated with 30-day mortality risk, but a significant interaction was observed between MCS use and lower LVEF, indicating a lower mortality risk in patients with a LVEF ≤ 20% treated with MCS. This could imply that use of MCS offers a net-benefit (e.g. expected benefit higher than risk of complications) in patients with a severely reduced LVEF. Two factors might contribute to explain this finding: First, in a severely reduced LVEF, the cardiac component is most likely the main factor driving outcome. Hence, MCS, which specifically addresses this issue by providing cardiac output support until native heart recovery or durable replacement therapy, is relatively more effective. Second, the more efficient and effective MCS is in supporting organ perfusion and bridging to cardiac recovery (and MCS explant) the less relevant any impact of MCS complications are likely to become. Thus, in this subpopulation of patients with non-ischaemic CS, the potential benefits of MCS usage may outweigh the associated risks of complications. These hypotheses warrant more in-depth exploration in the future research endeavours within this field.

These findings may inform the clinical decision on when to use MCS in patients presenting with non-ischaemic CS, especially when embedded within the case-based discussions of a well-organised “Shock Team” [36, 37]. Aside from potentially guiding the use of MCS in clinical practice, these results yield a rationale for using severely reduced LVEF as an inclusion criterion for randomised MCS trials. In the current RCTs testing MCS devices, LVEF is only used in the DanGer trial as an inclusion criterion [12], but not in others [13, 30, 38]. Future trials may opt to also include an enrolment criterion which reflects LV function.

### Limitations

The main strengths of this study are the use of a large, contemporary, international, multi-centre registry dedicated to the enrolment of patients with non-ischaemic CS. The main limitation of this study is the non-randomised retrospective design, so that a causal relation between risk predictors and outcome cannot be concluded.

Furthermore, the assessment of LVEF is susceptible to examiner- and centre-dependent subjectivity, especially when evaluated under clinically challenging conditions in intensive care units, emergency departments or catheterization laboratories with patients in a supine position. Whilst a centre-specific adjustment was conducted as a sensitivity analysis, revealing once more that there was no significant association between LVEF and 30-day mortality, but again a significant interaction between MCS use and LVEF ≤ 20%, it is crucial to underscore the following: this adjustment can reduce inter-observer variability, but it does not replace the valuable comparison of additional TTE measurements and invasively obtained haemodynamic data. It has been demonstrated that parameters, such as Cardiac Power Index, Cardiac Output, Cardiac Index and Stroke Volume, could potentially hold prognostic relevance in the context of cardiogenic shock [39]. Likewise, evaluating the prediction of afterload-related cardiac performance would be of interest [40]. However, these parameters are not adequately represented in this registry and should be the focus of future research endeavours dedicated to LVEF in non-ischaemic cardiogenic shock. Additionally, the lack of relevant data on co-existing valvular diseases, which could influence both baseline LVEF and subsequent treatment decisions, was not adequately represented in this registry.

Although the data were generated from different hospitals in different countries, all hospitals are large tertiary care centres with experience in treating patients with CS and with using MCS devices and all centres operate as part of a hub-and-spoke model. This may result in a higher prevalence of more severe CS and also higher use of MCS per se. In addition, the use of MCS in practice is a selective process in which patients with a higher physiological reserve are more likely to be treated with MCS devices, resulting in selection bias which might have influenced our results. Therefore, the generalizability of these data may be limited.

## Conclusion

This retrospective, multicenter, international study presents novel insights into LVEF assessment in patients with non-ischaemic CS. Although LVEF was not a predictor of 30-day mortality risk, there was a significant interaction between MCS use, LVEF and mortality, indicating a possible lower mortality risk with MCS use only in patients with a severely reduced LVEF. This may propose the utilisation of LVEF as an adjunctive criterion for guiding MCS therapy in non-ischaemic CS, and might also inform the design of randomised controlled MCS trials.

### Supplementary Information

Below is the link to the electronic supplementary material.

Supplementary file1 (DOCX 2714 kb)

## Abbreviations

AMI: Acute myocardial infarction
CS: Cardiogenic shock
LV: Left ventricular
LVEF: Left ventricular ejection fraction
MCS: Mechanical circulatory support
OHCA: Out-of-hospital cardiac arrest
pLVAD: Percutaneous left ventricular assist device
RCT: Randomised controlled trail
SCAI: Society for Cardiovascular Angiography and Interventions
TTE: Transthoracic echocardiogram
VA-ECMO: Veno-arterial extracorporeal membrane oxygenation
