# Supplementary material for: Association between left ventricular ejection fraction, mortality and use of mechanical circulatory support in patients with non-ischaemic cardiogenic shock
Source: Clin Res Cardiol. 2023 Nov 20;113(4):570–80. doi: 10.1007/s00392-023-02332-y (PMC10954940; doi:10.1007/s00392-023-02332-y)

***SUPPLEMENTARY APPENDIX***

***Supplementary figure 1***. Kaplan–Meier curves of the study cohort comparing patients with non-ischaemic cardiogenic shock and LVEF >20% versus ≤20%.


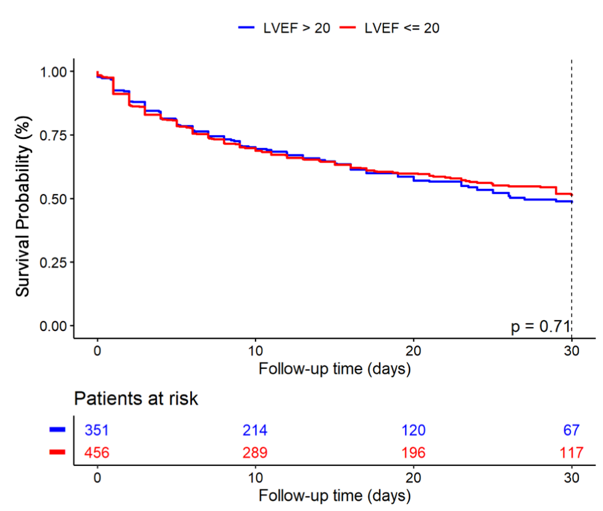


LVEF: Left ventricular ejection fraction.

***Supplementary figure 2.*** Boxplot of left ventricular ejection fraction (%) per centre cohort.


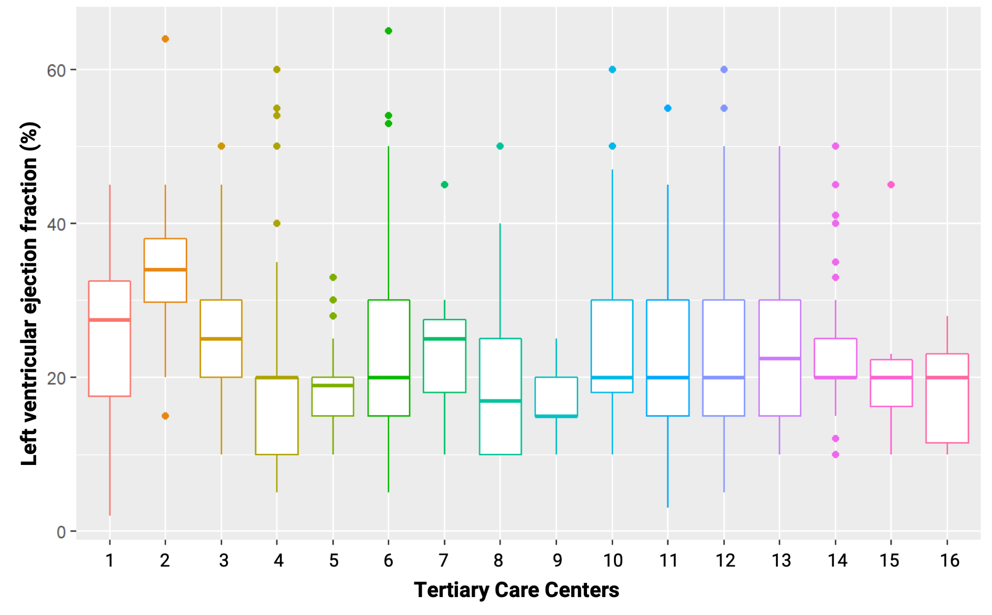

Supplement: Supplementary file 1 — Supplementary file1 (DOCX 2714 kb) [file 392_2023_2332_MOESM1_ESM.docx]
